# Colon-Derived Liver Metastasis, Colorectal Carcinoma, and Hepatocellular Carcinoma Can Be Discriminated by the Ca2+-Binding Proteins S100A6 and S100A11

**DOI:** 10.1371/journal.pone.0003767

**Published:** 2008-12-02

**Authors:** Christian Melle, Günther Ernst, Bettina Schimmel, Annett Bleul, Ferdinand von Eggeling

**Affiliations:** Core Unit Chip Application, Institute of Human Genetics and Anthropology, Medical Faculty at the Friedrich Schiller University, Jena, Germany; Cleveland Clinic, United States of America

## Abstract

**Background:**

It is unknown, on the proteomic level, whether the protein patterns of tumors change during metastasis or whether markers are present that allow metastases to be allocated to a specific tumor entity. The latter is of clinical interest if the primary tumor is not known.

**Methodology/Principal Findings:**

In this study, tissue from colon-derived liver metastases (n = 17) were classified, laser-microdissected, and analysed by ProteinChip arrays (SELDI). The resulting spectra were compared with data for primary colorectal (CRC) and hepatocellular carcinomas (HCC) from our former studies. Of 49 signals differentially expressed in primary HCC, primary CRC, and liver metastases, two were identified by immunodepletion as S100A6 and S100A11. Both proteins were precisely localized immunohistochemically in cells. S100A6 and S100A11 can discriminate significantly between the two primary tumor entities, CRC and HCC, whereas S100A6 allows the discrimination of metastases and HCC.

**Conclusions:**

Both identified proteins can be used to discriminate different tumor entities. Specific markers or proteomic patterns for the metastases of different primary cancers will allow us to determine the biological characteristics of metastasis in general. It is unknown how the protein patterns of tumors change during metastasis or whether markers are present that allow metastases to be allocated to a specific tumor entity. The latter is of clinical interest if the primary tumor is not known.

## Introduction

Distant metastases are the principal causes of death in patients with colorectal carcinoma (CRC). A common site of metastases derived from CRC is the liver.[1] The underlying mechanisms of liver metastasis of CRC are not fully understood, but metastases are at least involved in tumor initiation and promotion, uncontrolled proliferation, angiogenesis, invasion, intra- and extravasation, and colony formation at the liver site.[2], [3] The analysis of the expression of a single protein is not practical because these processes seem to be induced by the altered expression of several different proteins. Proteomic approaches are practical in the global analysis of altered protein patterns, in which diverse mass spectrometry (MS)-based methods are used for these kinds of high-throughput analyses.[4], [5] In this context, surface-enhanced laser desorption/ionization (SELDI) is a proteomic high-throughput technique that uses chromatographic surfaces that are able to retain proteins depending on their physico-chemical properties, followed by direct analysis via time-of-flight mass spectrometry (TOF-MS).[6] A multitude of studies using ProteinChip technology have been carried out to establish the protein profiles of biological fluids, especially serum samples.[7]–[9] Because this technique demands only a small amount of sample, it can be used for small biopsies or microdissected tissues, which produce the homogeneous tissue samples typically used in cancer research. The separation of functional tissue areas can be achieved by laser-based microdissection (for review see [10]). When laser microdissection was first introduced as a novel preparation technique in 1998, the challenge was to prove that reliable results could be achieved by selecting defined small amounts of isolated cells from complex tissue sections.[11] Since then numerous applications has been published in different fields and has proven its necessity.[12] Microdissected tissue material free from contaminating and unwanted tissue components is extremely important for the production of clean data for biomarker identification in cancer diagnostics and in determining the clonal heterogeneity of tumors. We have shown in a previous study that the detection of differentially expressed proteins was only possible in pure microdissected samples.[13] Laser-based microdissection has previously been combined with ProteinChip technology to identify protein markers in several cancer types.[14]–[16]

The aim of this study was to analyse the protein patterns of liver metastases derived from CRC (MTS) and detect biologically and diagnostically relevant signals. We wanted to analyze whether it is possible to draw conclusions from the proteome of the MTS on the origin/localization of the primary tumor.

## Materials and Methods

### Laser microdissection of tissue sections

All 17 human samples from liver metastases derived from CRC (MTS) were obtained after surgical resection at the Department of General and Visceral Surgery of the Friedrich Schiller University, Jena. They were collected fresh, snap frozen in liquid nitrogen, and stored at −80°C. Primary tumor specimens were categorized according to the WHO classification. Most of these tumors were classified as pT2 and pT3.

Laser microdissection was performed with a laser microdissection and pressure catapulting microscope (LMPC; Palm, Bernried, Germany) as previously described.[17] Briefly, we microdissected native air-dried cryostat tissue sections of approximately 3000–5000 cells, each in a maximum of 20–30 min. Proteins were extracted in 10 µL lysis buffer (100 mM Na-phosphate [pH 7.5], 5 mM EDTA, 2 mM MgCl_2_, 3 mM 2-β-mercaptoethanol, 0.1% CHAPS, 500 µM leupeptin, and 0.1 mM PMSF) for 30 min on ice. After centrifugation (15 min; 15,000 rpm) the supernatant was immediately analysed or frozen in liquid nitrogen for a maximum of one day.

### Profiling microdissected liver-localized metastases

The protein lysates from microdissected metastatic tissues were analysed on strong anion exchange arrays (Q10) (Bio-Rad), as previously described.[17] In brief, Q10 array spots were pre-incubated in a washing/loading buffer containing 100 mM Tris buffer (pH 8.5) and 0.02% Triton X-100. Then 2 µL sample aliquots were applied to the ProteinChip Arrays, which were incubated at room temperature for 90 min in a humidified chamber. After the samples had been washed three times with the fresh buffer and twice with water, 2×0.5 µL of sinapinic acid (saturated solution in 0.5% TFA/50% acetonitrile) were applied. Mass analysis was performed with a ProteinChip Reader (Series 4000; Ciphergen Biosystems Inc., Fremont, CA), according to an automated data collection protocol.

### Immunodepletion assay

Two microlitres of anti-S100A6 antibody (ab 141; Swant, Bellinzona, CH) or a specific antibody directed against S100A11 (rabbit polyclonal; Protein Tech Group, IL) were incubated with 10 µL of protein A–agarose (Sigma, St Louis, MO) for 15 min on ice. A pellet was generated by centrifugation and the supernatant was discarded. The pellet was washed twice with buffer containing 20 mM Hepes (pH 7.8), 25 mM KCl, 5 mM MgCl_2_, 0.1 mM EDTA, and 0.05% NP-40. Then, lysate (5 µL) from the microdissected tissue was incubated with this pellet for 45 min on ice. As a negative control, 5 µL of the lysate was incubated for 45 min on ice with protein A–agarose without the specific antibody. After incubation, the samples were cleared by centrifugation and 3 µL of each supernatant (immunodepleted sample) was analysed on the ProteinChip Arrays (Q10, BioRad).

### Immunohistochemistry

Cryostat sections (8 µm) of MTS-containing tissue (n = 5) or CRC tissue (n = 5) were placed on slides, air-dried for about 60 min at 20°C, and fixed in paraformaldehyde, as described previously.[18] After fixation, the slides were treated in a microwave at 80 watts (3×3 min) in 10 mM citric acid (pH 6.0) to inhibit endogenous peroxidase activity. They were then rinsed twice with Tris-buffered saline (TBS; pH 7.4), and incubated overnight at 4°C in a humidified chamber with the corresponding primary anti-S100A6 antibody or anti-S100A11 antibody. The slides were rinsed three times, for 10 min each, in TBS. The Vectastain Elite ABC kit (Vector Laboratories, Burlingame, CA) and the Jenchrom pxbl-kit (MoBiTec, Göttingen, Germany) were used according to the manufacturers' instructions to visualize the antibodies. Negative controls were incubated with only the labelled secondary antibody. Sections cut in parallel to the immunohistochemically treated sections were stained with haematoxylin–eosin for better identification of the different tissue areas. Immunohistochemical staining was evaluated by a pathologist.

### Statistical Analyses

Mass spectra from ProteinChip arrays were normalized to a total ion current and cluster analysis of the detected signals was performed. The respective *P* values for MTS, CRC,[19] and HCC [17] were determined with the CiphergenExpress program (version 3.0; Ciphergen Biosystems Inc.). To calculate the *P* values, normalized spectra with signals in the range of 2.5–200 kDa, with a signal-to-noise ratio (S/N) of at least 10, were selected and analysed with the Mann–Whitney U test for non-parametric data sets, and the Kruskal–Wallis test. Receiver operating characteristic (ROC) curves [20] were constructed for S100A6 and S100A11 expression data derived CRC, MTS or HCC, respectively by plotting sensitivity versus 1-specificity (CiphergenExpress 3.0).

## Results

### Proteomic analysis of microdissected tissues from liver metastases derived from CRC, primary CRC, and HCC by SELDI–MS

**Figure 1:**
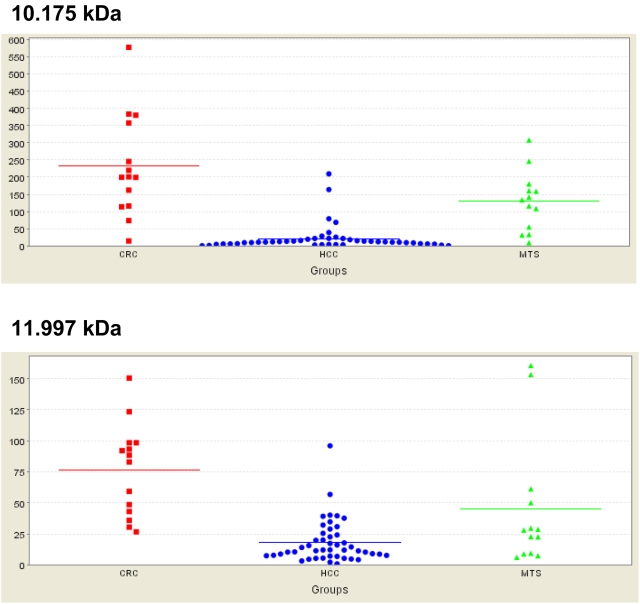
Distribution of the intensities of peaks expressed significantly differently in liver metastases derived from colorectal carcinoma (MTS), colorectal carcinoma tumor (CRC), and hepatocellular carcinoma (HCC) tumor margins. *Upper panel*: Distribution of the intensities of the 10.175 kDa signal. *Bottom panel*: Distribution of the intensities of the 11.997 kDa signal. The spectra were obtained using Q10 arrays. X-axis indicates the sample groups, Y-axis the intensity (μA).

For this study, microdissected tissue probes containing about 3000–5000 cells each were successfully dissected from 17 liver metastases derived from colorectal cancer (MTS) by an experienced pathologist. All protein lysates were applied to strong anion exchanger Q10 ProteinChip Arrays and analysed individually by SELDI–MS on a ProteinChip Reader Series 4000 instrument. The spectra generated from the MTS were compared with specific spectra derived from primary CRC (n = 14) and HCC (n = 46), which were generated as described previously [17], [19], to detect any distinguishing protein signals. In the range of 2.5–200 kDa, up to 372 peaks were detected with normalized intensities. After evaluation with the CiphergenExpress program, many significantly different signals (n = 49) were detected for MTS, CRC, and HCC (Table S1). Among these, the peak masses with markedly low *P* values were selected for further identification and characterization. The signals at 10.175 kDa (*P* = 3.00×10^−9^) and 11.997 kDa (*P* = 1.82×10^−6^) were significantly upregulated in both MTS and CRC compared with samples derived from HCC (Figure 1). The signal with a molecular mass of 10.175 kDa was the most significant single signal capable of discriminating between the two sample groups in this analysis. The 11.997 kDa signal was ranked in 12th position (Table S1). Representative examples of SELDI–MS spectra of MTS, CRC, and HCC are shown in Figure S1.

### Identification of differentially expressed protein peaks

**Figure 2:**
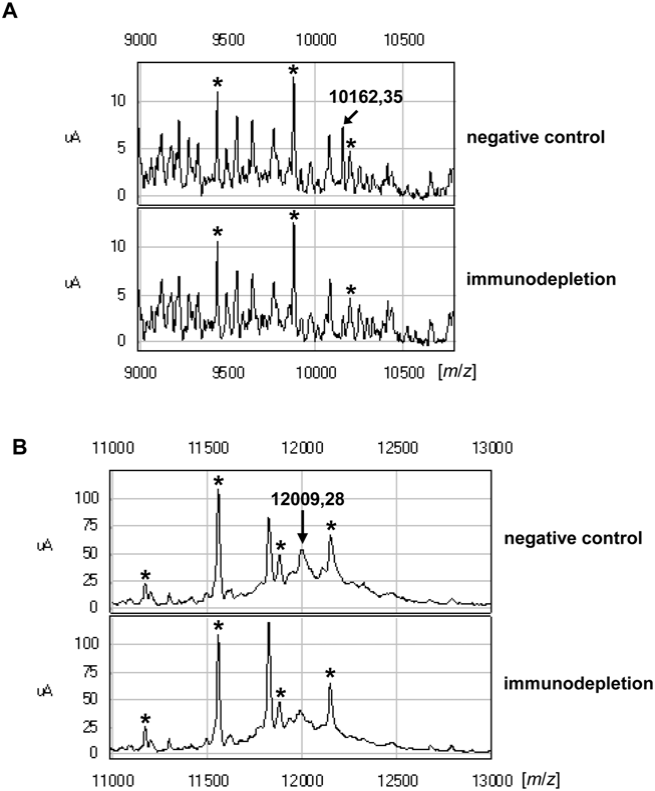
Immunodepletion assays of S100A6 and S100A11. Normalized ProteinChip® array profiles of metastatic tissue show that the peaks representing S100A6 (A, 10.162 kDa) or S100A11 (B, 12.009 kDa) were detectable in the negative controls but only with decreased intensity with the corresponding depleted probes. Reference peaks that were not influenced by immunodepletion are labelled with asterisks.

The interesting proteins with molecular masses of 10.175 kDa and 11.997 kDa, which corresponded very well to the Ca^2+^-binding proteins S100A6 (NCBI NP_055439) and S100A11 (NCBI NP_005611), respectively, were also detected by ProteinChip technology, as has been described by ours and other groups [17], [19], [21]. To confirm that S100A6 and S100A11 match the differentially expressed peaks observed at 10.175 kDa and 11.997 kDa, respectively, in this protein profiling analysis, immunodepletion assays were carried out using microdissected MTS tissue as the starting material. Analysis of the supernatants of the immunodepletion assays by Q10 arrays showed that the peaks corresponding to S100A6 and S100A11 were significantly reduced. In the negative controls without specific antibodies, the peaks were clearly detectable (Figure 2). The identification of other differentially expressed proteins is in progress.

### Characterization of the differentially expressed protein signals

**Figure 3:**
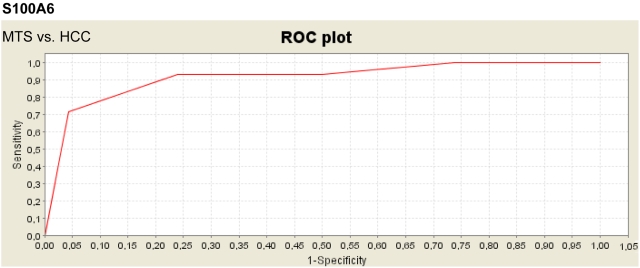
ROC curve of S100A6 in liver metastases derived from colorectal carcinoma (MTS) and in hepatocellular carcinoma (HCC). S100A6 is significantly upregulated in MTS.

**Figure 4:**
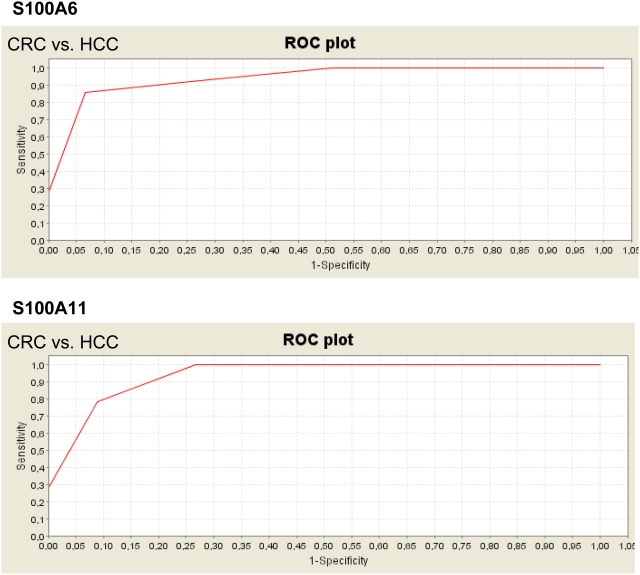
ROC curves of S100A6 and S100A11 in colorectal carcinoma (CRC) and in hepatocellular carcinoma (HCC). Both proteins are significantly upregulated in CRC.

To assess the impact of S100A6 and S100A11 as discriminatory signals for different tumorous tissue samples, we compared the spectra derived from different sample groups in individual assays. We found that S100A6 was significantly upregulated in CRC and MTS compared with HCC (*P* = 2.81×10^−6^) (Figure 3). Hence, S100A again ranked first as the most significant signal (Table S2). An analysis of CRC and HCC showed that S100A6 (*P* = 2.62×10^−7^) and S100A11 (*P* = 5.54×10^−7^) were both significantly upregulated in the samples derived from CRC (Figure 4). In this analysis, S100A6 and S100A11 ranked in third place and fourth place, respectively, in the most significant signals (Table S3). Interestingly, neither S100A6 nor S100A11 were significantly differentially expressed in MTS compared with CRC (Table S4).

### Localization of S100A6 and S100A11 in tissue

**Figure 5:**
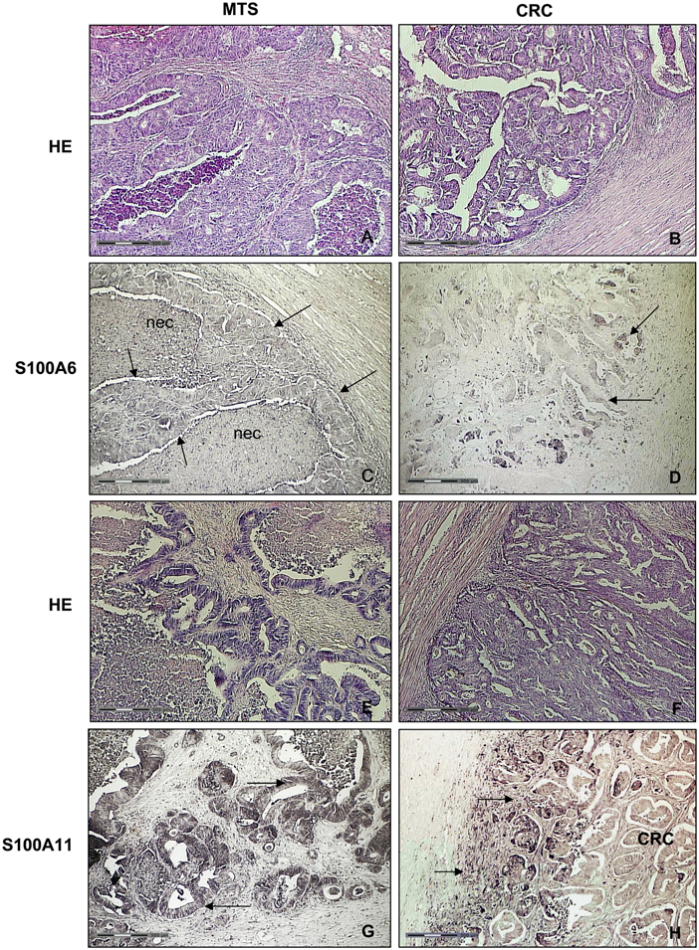
Immunohistochemical analysis of S100A6 and S100A11 and corresponding H&E sections. (A) and (B) H&E section from liver metastases derived from colorectal carcinoma (MTS) and colorectal carcinoma (CRC). (C) Corresponding section to (A) immunostained for S100A6 using a specific antibody. A positive immunohistochemical reaction was detectable in the MTS (labeled with arrows) and in adjacent necrotic tissue (nec). The surrounding liver tissue was negative. (D) Corresponding section to (B). Scattered immunoreactive tumor cell complexes (labeled with arrows) at the tumor periphery of a colorectal carcinoma (CRC) detected with anti-S100A6 antibody. (E) and (F) H&E section from liver metastases derived from colorectal carcinoma (MTS) and colorectal carcinoma (CRC). (G) Corresponding section to (E). S100A11-positive tumor cell complex (labeled with an arrow) in MTS. (H) Corresponding section to (F). Strong S100A11 immunoreactivity in CRC tumor cell complexes (labeled with arrows). Immunoreactivity against S100A11 was stronger in the tumor margin compared to central tumor area.

To confirm their identification and in particular to localize S100A6 and S100A11 in tissue sections, we assessed their expression in MTS and in primary CRC by immunohistochemistry using specific antibodies. All these tissues showed a positive reaction to the antibodies directed against S100A6 or S100A11 (Figure 5). In contrast to these findings, only very poor signals were detectable in the tissue surrounding the MTS (Figure 5A and D). Interestingly, the immunoreactivity of the tumor cell complexes was stronger at the tumor margin than that in the central tumor area in CRC, when we used the specific antibody directed against S100A6 (Figure 5E). Consistent with the SELDI analysis of HCC, neither signal was detectable by immunohistochemistry (data not shown).

## Discussion

Liver metastasis of CRC is a major reason for the poor prognosis of patients. An improved understanding of the molecular and cellular mechanisms underlying metastasis would contribute greatly to its early detection and treatment. The initiation of MTS affects the expression of multiple proteins.[22]–[24] The identification of proteins that are characteristic of metastasis might allow the discrimination of different tumor entities. To address this challenge, specific proteomic approaches have been used that focus on the complex analysis of protein levels in metastases using cell lines.[2527] Such altered expression patterns have been detected for cytokeratin 18, tissue transglutaminase, Rho GDP-dissociation inhibitor 1, fibroblast-type tropomyosin, interleukin-18, annexin I, disulfide isomerase, heat shock protein 60, peroxiredoxin 1, chlorine intracellular channel protein 1, and creatine kinase B chain, as well as for some ribosomal proteins. Since the early 1990s, a number of studies have investigated, with genomic approaches, tissues derived directly from both primary tumors and organs involved in metastasis.[28]–[31] This is, to the best of our knowledge, the first study to use a proteomic approach in a comparative investigation of tissues derived from different metastasising primary tumors and in the identification of proteins that can discriminate between these tumor types and between tumors and metastases. In contrast to the majority of SELDI–MS-based studies, we identified significantly differentially expressed proteins. The Ca^2+^-binding proteins identified here, S100A6 and S100A11, can distinguish very clearly between MTS, primary CRC, and primary HCC, as well as between CRC and HCC. A number of additional signals were detected that discriminate between MTS and CRC, but S100A6 and S100A11 do not. The identification of specific signals that can distinguish between metastases and the primary tumor is in progress. Until now, only two small studies have comparatively assessed the expression of S100A6 in human colorectal mucosa, primary colorectal adenocarcinomas, and liver metastases using a specific western blot analysis [32], [33]. In contrast, we analysed an extended number of samples using a hypothesis-free proteomic approach and thereby detected and identified S100A6 as a factor with the potential to discriminate between primary HCC and MTS. S100A6 and S100A11 belong to the group of S100 proteins involved in the Ca^2+^ signalling network, and regulate intracellular activities such as cell growth and motility, cell-cycle progression, transcription, and cell differentiation.[34], [35] Both S100A6 and S100A11 have been observed in several epithelial tumors and are linked to metastasis.[19], [32], [36], [37]

In this study, we have demonstrated the potential of SELDI–MS in characterizing metastasis in terms of protein profiles and in discriminating between different tumor entities. In future, it would be very interesting to assess and compare the protein profiles of metastases derived from different types of metastasising tumors. We might expect to find a panel of protein signals or a “metastatic protein profile” that is common to all metastatic tissues. This panel will presumably contain proteins involved in the coordination of metastatic processes. Although neither S100A6 nor S100A11 can discriminate between MTS and the corresponding primary CRC, they can discriminate between primary CRC and primary HCC. Perhaps more importantly, S100A6 is a potential candidate to discriminate between MTS and primary HCC. The discrimination of primary HCC and its metastases located in the liver is presently complicated and afflicted with difficulties.[38] Therefore, S100A6 might provide some resolution of this problem.

## Supporting Information

Figure S1 Representative examples of SELDI-TOF MS spectra of liver metastases derived from colorectal carcinoma (MTS), colorectal carcinoma (CRC), and hepatocellular carcinoma (HCC). Data are obtained using Q10 arrays. The peaks of interest at 10.175 kDa and 11.997 kDa are marked with frames.
(0.18 MB DOC)

Table S1 (0.07 MB DOC)

Table S2 (0.05 MB DOC)

Table S3 (0.06 MB DOC)

Table S4 (0.04 MB DOC)

## References

[1] Nakamura S, Suzuki S, Baba S (1997). Resection of liver metastases of colorectal carcinoma.. World J Surg.

[2] Weiss L (2000). Metastasis of cancer: a conceptual history from antiquity to the 1990s.. Cancer Metastasis Rev.

[3] Ridley A (2000). Molecular switches in metastasis.. Nature.

[4] Pan S, Zhang H, Rush J, Eng J, Zhang N (2005). High throughput proteome screening for biomarker detection.. Mol Cell Proteomics.

[5] Drake RR, Schwegler EE, Malik G, Diaz J, Block T (2006). Lectin capture strategies combined with mass spectrometry for the discovery of serum glycoprotein biomarkers.. Mol Cell Proteomics.

[6] Tang N, Tornatore P, Weinberger SR (2004). Current developments in SELDI affinity technology.. Mass Spectrom Rev.

[7] Paradis V, Degos F, Dargere D, Pham N, Belghiti J (2005). Identification of a new marker of hepatocellular carcinoma by serum protein profiling of patients with chronic liver diseases.. Hepatology.

[8] Li Y, Dang TA, Shen J, Perlaky L, Hicks J (2006). Identification of a plasma proteomic signature to distinguish pediatric osteosarcoma from benign osteochondroma.. Proteomics.

[9] Ward DG, Suggett N, Cheng Y, Wei W, Johnson H (2006). Identification of serum biomarkers for colon cancer by proteomic analysis.. Br J Cancer.

[10] von Eggeling F, Melle C, Ernst G (2007). Microdissecting the proteome.. Proteomics.

[11] Schütze K, Lahr G (1998). Identification of expressed genes by laser-mediated manipulation of single cells.. Nat Biotechnol.

[12] Schütze K, Niyaz Y, Stich M, Buchstaller A (2007). Noncontact laser microdissection and catapulting for pure sample capture.. Methods Cell Biol.

[13] Melle C, Ernst G, Schimmel B, Bleul A, Thieme H (2005). Discovery and identification of alpha-defensins as low abundant, tumor-derived serum markers in colorectal cancer.. Gastroenterology.

[14] Cheung PK, Woolcock B, Adomat H, Sutcliffe M, Bainbridge TC (2004). Protein profiling of microdissected prostate tissue links growth differentiation factor 15 to prostate carcinogenesis.. Cancer Res.

[15] Melle C, Ernst G, Schimmel B, Bleul A, Koscielny S (2003). Biomarker Discovery and Identification in Laser Microdissected Head and Neck Squamous Cell Carcinoma with ProteinChip(R) Technology, Two-dimensional Gel Electrophoresis, Tandem Mass Spectrometry, and Immunohistochemistry.. Mol Cell Proteomics.

[16] Guedj N, Dargere D, Degos F, Janneau JL, Vidaud D (2006). Global proteomic analysis of microdissected cirrhotic nodules reveals significant biomarkers associated with clonal expansion.. Lab Invest.

[17] Melle C, Ernst G, Scheibner O, Kaufmann R, Schimmel B (2007). Identification of Specific Protein Markers in Microdissected Hepatocellular Carcinoma.. J Proteome Res.

[18] Melle C, Ernst G, Schimmel B, Bleul A, Thieme H (2005). Discovery and identification of alpha-defensins as low abundant, tumor-derived serum markers in colorectal cancer.. Gastroenterology.

[19] Melle C, Ernst G, Schimmel B, Bleul A, Mothes H (2006). Different expression of calgizzarin (S100A11) in normal colonic epithelium, adenoma and colorectal carcinoma.. International Journal of Oncology.

[20] Raslich MA, Markert RJ, Stutes SA (2007). Selecting and interpreting diagnostic tests.. Biochemia Medica.

[21] Melle C, Ernst G, Schimmel B, Bleul A, Koscielny S (2004). A Technical Triade for Proteomic Identification and Characterization of Cancer Biomarkers.. Cancer Res.

[22] Mc DS, Chaudhry V, Mansilla-Soto J, Zeng ZS, Shu WP (1999). Metastatic and non-metastatic colorectal cancer (CRC) cells induce host metalloproteinase production in vivo.. Clin Exp Metastasis.

[23] Tzimas GN, Chevet E, Jenna S, Nguyen DT, Khatib AM (2005). Abnormal expression and processing of the proprotein convertases PC1 and PC2 in human colorectal liver metastases.. BMC Cancer.

[24] Ueda J, Semba S, Chiba H, Sawada N, Seo Y (2007). Heterogeneous expression of claudin-4 in human colorectal cancer: decreased claudin-4 expression at the invasive front correlates cancer invasion and metastasis.. Pathobiology.

[25] Jiang D, Ying W, Lu Y, Wan J, Zhai Y (2003). Identification of metastasis-associated proteins by proteomic analysis and functional exploration of interleukin-18 in metastasis.. Proteomics.

[26] Cai Z, Chiu JF, He QY (2004). Application of proteomics in the study of tumor metastasis.. Genomics Proteomics Bioinformatics.

[27] Kreunin P, Yoo C, Urquidi V, Lubman DM, Goodison S (2007). Differential expression of ribosomal proteins in a human metastasis model identified by coupling 2-D liquid chromatography and mass spectrometry.. Cancer Genomics Proteomics.

[28] Finkelstein SD, Sayegh R, Christensen S, Swalsky PA (1993). Genotypic classification of colorectal adenocarcinoma. Biologic behavior correlates with K-ras-2 mutation type.. Cancer.

[29] Sanchez-Cespedes M, Esteller M, Hibi K, Cope FO, Westra WH (1999). Molecular detection of neoplastic cells in lymph nodes of metastatic colorectal cancer patients predicts recurrence.. Clin Cancer Res.

[30] Khan ZA, Jonas SK, Le-Marer N, Patel H, Wharton RQ (2000). P53 mutations in primary and metastatic tumors and circulating tumor cells from colorectal carcinoma patients.. Clin Cancer Res.

[31] Conzelmann M, Linnemann U, Berger MR (2005). Molecular detection of clinical colorectal cancer metastasis: how should multiple markers be put to use?. Int J Colorectal Dis.

[32] Komatsu K, Kobune-Fujiwara Y, Andoh A, Ishiguro S, Hunai H (2000). Increased expression of S100A6 at the invading fronts of the primary lesion and liver metastasis in patients with colorectal adenocarcinoma.. Br J Cancer.

[33] Komatsu K, Murata K, Kameyama M, Ayaki M, Mukai M (2002). Expression of S100A6 and S100A4 in matched samples of human colorectal mucosa, primary colorectal adenocarcinomas and liver metastases.. Oncology.

[34] Schafer BW, Heizmann CW (1996). The S100 family of EF-hand calcium-binding proteins: functions and pathology.. Trends Biochem Sci.

[35] Donato R (2001). S100: a multigenic family of calcium-modulated proteins of the EF-hand type with intracellular and extracellular functional roles.. Int J Biochem Cell Biol.

[36] Maelandsmo GM, Florenes VA, Mellingsaeter T, Hovig E, Kerbel RS (1997). Differential expression patterns of S100A2, S100A4 and S100A6 during progression of human malignant melanoma.. Int J Cancer.

[37] Yao R, Davidson DD, Lopez-Beltran A, MacLennan GT, Montironi R (2007). The S100 proteins for screening and prognostic grading of bladder cancer.. Histol Histopathol.

[38] Helmberger TK, Laubenberger J, Rummeny E, Jung G, Sievers K (2002). MRI characteristics in focal hepatic disease before and after administration of MnDPDP: discriminant analysis as a diagnostic tool.. Eur Radiol.

