# Supplementary material for: Colon-Derived Liver Metastasis, Colorectal Carcinoma, and Hepatocellular Carcinoma Can Be Discriminated by the Ca2+-Binding Proteins S100A6 and S100A11
Source: PLoS One. 2008 Dec 2;3(12):e3767. doi: 10.1371/journal.pone.0003767 (PMC2585013; doi:10.1371/journal.pone.0003767)
Supplement: Figure S1 — Representative examples of SELDI-TOF MS spectra of liver metastases derived from colorectal carcinoma (MTS), colorectal carcinoma (CRC), and hepatocellular carcinoma (HCC). Data are obtained using Q10 arrays. The peaks of interest at 10.175 kDa and 11.997 kDa are marked with frames. (0.18 MB DOC) [file pone.0003767.s001.doc]

**Figure S1.**

Representative examples of SELDI-TOF MS spectra of liver metastases derived from colorectal carcinoma (MTS), colorectal carcinoma (CRC), and hepatocellular carcinoma (HCC). Data are obtained using Q10 arrays. The peaks of interest at 10.175 kDa and 11.997 kDa are marked with frames.

**CRC**

**HCC**

**MTS**

Table 1: Significantly different signals that distinguish tissues from liver metastases derived from colorectal carcinoma (MTS), colorectal carcinoma (CRC), and hepatocellular carcinoma (HCC), detected on Q10 arrays. The signals representing the subsequently identified S100A6 and S100A11 are shown in bold.

Table 2: Significantly different signals that distinguish tissues from liver metastases derived from colorectal carcinoma (MTS) and hepatocellular carcinoma (HCC), detected on Q10 arrays. The signal corresponding to S100A6 is shown in bold.

Table 3: Significantly different signals that distinguish colorectal carcinoma (CRC) and hepatocellular carcinoma (HCC), detected on Q10 arrays. The signals representing S100A6 and S100A11 are shown in bold.

Table 4: Significantly different signals which separate tissues from liver metastases derived from colorectal carcinoma (MTS) and colorectal carcinoma (CRC) detected on Q10 arrays.

Table 1.

| **Signal in** | **MW (kD)** | **P-value** |
| --- | --- | --- |
| MTS | 3.483 | 3.23x10^-2^ |
| CRC | 4.968 | 8.66x10^-4^ |
| CRC | 5.077 | 5.35x10^-7^ |
| HCC | 5.362 | 1.82x10^-7^ |
| MTS | 5.658 | 2.09x10^-3^ |
| CRC | 5.943 | 4.12x10^-2^ |
| HCC | 6.648 | 1.09x10^-3^ |
| HCC | 6.736 | 8.35x10^-4^ |
| HCC | 7.571 | 1.75x10^-5^ |
| HCC | 7.667 | 1.07x10^-4^ |
| HCC | 7.943 | 2.87x10^-3^ |
| CRC | 8.225 | 6.54x10^-6^ |
| CRC | 8.411 | 3.66x10^-2^ |
| HCC | 9.163 | 1.02x10^-4^ |
| HCC | 9.613 | 1.16x10^-7^ |
| HCC | 9.976 | 4.07x10^-6^ |
| CRC | **10.175** | 3.00x10^-9^ |
| CRC | 10.358 | 2.80x10^-7^ |
| CRC | 10.394 | 6.30x10^-8^ |
| MTS | 11.315 | 1.35x10^-2^ |
| MTS | 11.357 | 1.23x10^-2^ |
| CRC | 11.683 | 4.49x10^-2^ |
| CRC | **11.997** | 1.82x10^-6^ |
| HCC | 13.546 | 1.39x10^-3^ |
| MTS | 13.783 | 7.62x10^-3^ |
| MTS | 14.018 | 1.48x10^-2^ |
| HCC | 14.975 | 1.68x10^-7^ |
| HCC | 15.138 | 5.14x10^-6^ |
| HCC | 15.351 | 1.34x10^-3^ |
| HCC | 15.882 | 8.32x10^-5^ |
| HCC | 19.944 | 3.00x10^-9^ |
| HCC | 20.845 | 1.40x10^-8^ |
| HCC | 21.285 | 1.52x10^-6^ |
| HCC | 22.271 | 3.20x10^-5^ |
| CRC | 23.162 | 7.85x10^-3^ |
| CRC | 23.807 | 3.50x10^-6^ |
| CRC | 24.807 | 2.79x10^-5^ |
| MTS | 28.117 | 1.59x10^-3^ |
| CRC | 32.045 | 1.32x10^-4^ |
| HCC | 41.762 | 4.20x10^-8^ |
| HCC | 44.215 | 4.31x10^-5^ |
| HCC | 46.497 | 1.65x10^-2^ |
| HCC | 51.508 | 1.15x10^-3^ |
| MTS | 53.673 | 2.16x10^-2^ |
| HCC | 54.428 | 3.34x10^-7^ |
| HCC | 55.435 | 3.00x10^-9^ |
| CRC | 68.187 | 5.45x10^-3^ |
| MTS | 78.225 | 2.46x10^-5^ |
| CRC | 118.336 | 2.50x10^-2^ |

Table 2

| **Signal in** | **MW (kD)** | **P-value** |
| --- | --- | --- |
| MTS | 3.483 | 3.75x10^-2^ |
| MTS | 5.075 | 1.49x10^-4^ |
| HCC | 5.362 | 1.34x10^-5^ |
| HCC | 6.646 | 4.63x10^-3^ |
| HCC | 6.731 | 2.80x10^-3^ |
| HCC | 9.163 | 3.71x10^-3^ |
| HCC | 9.616 | 1.24x10^-5^ |
| HCC | 9.974 | 7.81x10^-5^ |
| MTS | **10.175** | 2.81x10^-6^ |
| MTS | 10.847 | 3.15x10^-2^ |
| HCC | 11.178 | 7.11x10^-3^ |
| HCC | 13.548 | 2.64x10^-3^ |
| HCC | 14.975 | 6.40x10^-3^ |
| HCC | 15.138 | 4.38x10^-3^ |
| HCC | 15.882 | 1.30x10^-2^ |
| HCC | 19.944 | 5.01x10^-5^ |
| HCC | 20.845 | 1.37x10^-5^ |
| MTS | 21.279 | 2.25x10^-4^ |
| HCC | 22.244 | 1.47x10^-4^ |
| MTS | 23.788 | 4.36x10^-3^ |
| MTS | 24.807 | 5.44x10^-3^ |
| MTS | 32.005 | 7.52x10^-3^ |
| HCC | 41.781 | 3.19x10^-4^ |
| HCC | 44.215 | 8.36x10^-3^ |
| HCC | 46.497 | 3.10x10^-2^ |
| HCC | 54.444 | 2.78x10^-4^ |
| HCC | 55.435 | 7.12x10^-6^ |
| MTS | 78.225 | 3.28x10^-3^ |
| MTS | 95.222 | 6.40x10^-3^ |
| MTS | 110.578 | 2.59x10^-2^ |

Table 3

| **Signal in** | **MW (kD)** | **P-value** |
| --- | --- | --- |
| CRC | 4.968 | 2.98x10^-4^ |
| CRC | 5.079 | 5.53x10^-6^ |
| HCC | 5.271 | 3.25x10^-2^ |
| HCC | 5.362 | 5.08x10^-6^ |
| HCC | 5.658 | 1.11x10^-3^ |
| CRC | 5.944 | 1.39x10^-2^ |
| HCC | 6.648 | 3.28x10^-3^ |
| HCC | 7.571 | 1.90x10^-5^ |
| HCC | 7.661 | 2.41x10^-5^ |
| HCC | 7.943 | 3.09x10^-3^ |
| CRC | 8.226 | 1.96x10^-6^ |
| CRC | 8.408 | 1.32x10^-2^ |
| HCC | 9.163 | 1.27x10^-4^ |
| HCC | 9.613 | 2.14x10^-6^ |
| HCC | 9.974 | 7.08x10^-5^ |
| CRC | **10.182** | 2.62x10^-7^ |
| CRC | 10.359 | 5.54x10^-7^ |
| CRC | 10.541 | 3.71x10^-2^ |
| HCC | 11.315 | 2.15x10^-2^ |
| CRC | **11.997** | 5.54x10^-7^ |
| HCC | 13.546 | 4.36x10^-3^ |
| HCC | 13.787 | 4.36x10^-3^ |
| HCC | 14.018 | 8.81x10^-3^ |
| HCC | 14.975 | 2.16x10^-7^ |
| HCC | 15.138 | 1.26x10^-5^ |
| HCC | 15.351 | 2.98x10^-4^ |
| HCC | 15.882 | 1.47x10^-4^ |
| HCC | 19.951 | 4.10x10^-8^ |
| HCC | 20.845 | 1.13x10^-5^ |
| HCC | 21.285 | 3.30x10^5^- |
| HCC | 22.276 | 3.88x10^-4^ |
| CRC | 23.166 | 2.54x10^-3^ |
| CRC | 23.801 | 8.83x10^-6^ |
| CRC | 24.807 | 9.09x10^-5^ |
| HCC | 28.003 | 4.85x10^-3^ |
| CRC | 32.045 | 4.45x10^-4^ |
| HCC | 41.781 | 4.05x10^-6^ |
| HCC | 51.559 | 2.37x10^-4^ |
| HCC | 54.396 | 1.13x10^-5^ |
| HCC | 55.435 | 1.98x10^-6^ |
| CRC | 68.188 | 1.16x10^-2^ |
| HCC | 82.182 | 3.30x10^-5^ |
| CRC | 118.336 | 6.08x10^-3^ |
| CRC | 172.524 | 5.85x10^-4^ |

Table 4

| **Signal in** | **MW (kD)** | **P-value** |
| --- | --- | --- |
| CRC | 4.966 | 3.76x10^-2^ |
| MTS | 5.655 | 1.26x10^-2^ |
| CRC | 8.208 | 4.10x10^-4^ |
| CRC | 10.356 | 2.40x10^-3^ |
| MTS | 10.845 | 1.82x10^-3^ |
| MTS | 11.308 | 3.39x10^-2^ |
| CRC | 11.681 | 2.75x10^-2^ |
| CRC | 11.835 | 4.60x10^-2^ |
| MTS | 14.024 | 4.16x10^-2^ |
| CRC | 23.162 | 6.94x10^-3^ |
| MTS | 47.952 | 2.51x10^-2^ |
| MTS | 51.431 | 3.62x10^-3^ |
| MTS | 53.641 | 6.11x10^-3^ |
| MTS | 94.521 | 8.90x10^-3^ |
