# Supplementary material for: Colon-Derived Liver Metastasis, Colorectal Carcinoma, and Hepatocellular Carcinoma Can Be Discriminated by the Ca2+-Binding Proteins S100A6 and S100A11
Source: PLoS One. 2008 Dec 2;3(12):e3767. doi: 10.1371/journal.pone.0003767 (PMC2585013; doi:10.1371/journal.pone.0003767)
Supplement: Table S1 — (0.07 MB DOC) [file pone.0003767.s002.doc]

Table S1. Significantly different signals that distinguish tissues from liver metastases derived from colorectal carcinoma (MTS), colorectal carcinoma (CRC), and hepatocellular carcinoma (HCC), detected on Q10 arrays. The signals representing the subsequently identified S100A6 and S100A11 are shown in bold.

| **Signal in** | **MW (kD)** | **P-value** |
| --- | --- | --- |
| MTS | 3.483 | 3.23x10^-2^ |
| CRC | 4.968 | 8.66x10^-4^ |
| CRC | 5.077 | 5.35x10^-7^ |
| HCC | 5.362 | 1.82x10^-7^ |
| MTS | 5.658 | 2.09x10^-3^ |
| CRC | 5.943 | 4.12x10^-2^ |
| HCC | 6.648 | 1.09x10^-3^ |
| HCC | 6.736 | 8.35x10^-4^ |
| HCC | 7.571 | 1.75x10^-5^ |
| HCC | 7.667 | 1.07x10^-4^ |
| HCC | 7.943 | 2.87x10^-3^ |
| CRC | 8.225 | 6.54x10^-6^ |
| CRC | 8.411 | 3.66x10^-2^ |
| HCC | 9.163 | 1.02x10^-4^ |
| HCC | 9.613 | 1.16x10^-7^ |
| HCC | 9.976 | 4.07x10^-6^ |
| CRC | **10.175** | 3.00x10^-9^ |
| CRC | 10.358 | 2.80x10^-7^ |
| CRC | 10.394 | 6.30x10^-8^ |
| MTS | 11.315 | 1.35x10^-2^ |
| MTS | 11.357 | 1.23x10^-2^ |
| CRC | 11.683 | 4.49x10^-2^ |
| CRC | **11.997** | 1.82x10^-6^ |
| HCC | 13.546 | 1.39x10^-3^ |
| MTS | 13.783 | 7.62x10^-3^ |
| MTS | 14.018 | 1.48x10^-2^ |
| HCC | 14.975 | 1.68x10^-7^ |
| HCC | 15.138 | 5.14x10^-6^ |
| HCC | 15.351 | 1.34x10^-3^ |
| HCC | 15.882 | 8.32x10^-5^ |
| HCC | 19.944 | 3.00x10^-9^ |
| HCC | 20.845 | 1.40x10^-8^ |
| HCC | 21.285 | 1.52x10^-6^ |
| HCC | 22.271 | 3.20x10^-5^ |
| CRC | 23.162 | 7.85x10^-3^ |
| CRC | 23.807 | 3.50x10^-6^ |
| CRC | 24.807 | 2.79x10^-5^ |
| MTS | 28.117 | 1.59x10^-3^ |
| CRC | 32.045 | 1.32x10^-4^ |
| HCC | 41.762 | 4.20x10^-8^ |
| HCC | 44.215 | 4.31x10^-5^ |
| HCC | 46.497 | 1.65x10^-2^ |
| HCC | 51.508 | 1.15x10^-3^ |
| MTS | 53.673 | 2.16x10^-2^ |
| HCC | 54.428 | 3.34x10^-7^ |
| HCC | 55.435 | 3.00x10^-9^ |
| CRC | 68.187 | 5.45x10^-3^ |
| MTS | 78.225 | 2.46x10^-5^ |
| CRC | 118.336 | 2.50x10^-2^ |
