# Supplementary material for: Colon-Derived Liver Metastasis, Colorectal Carcinoma, and Hepatocellular Carcinoma Can Be Discriminated by the Ca2+-Binding Proteins S100A6 and S100A11
Source: PLoS One. 2008 Dec 2;3(12):e3767. doi: 10.1371/journal.pone.0003767 (PMC2585013; doi:10.1371/journal.pone.0003767)
Supplement: Table S3 — (0.06 MB DOC) [file pone.0003767.s004.doc]

Table S3. Significantly different signals that distinguish colorectal carcinoma (CRC) and hepatocellular carcinoma (HCC), detected on Q10 arrays. The signals representing S100A6 and S100A11 are shown in bold.

| **Signal in** | **MW (kD)** | **P-value** |
| --- | --- | --- |
| CRC | 4.968 | 2.98x10^-4^ |
| CRC | 5.079 | 5.53x10^-6^ |
| HCC | 5.271 | 3.25x10^-2^ |
| HCC | 5.362 | 5.08x10^-6^ |
| HCC | 5.658 | 1.11x10^-3^ |
| CRC | 5.944 | 1.39x10^-2^ |
| HCC | 6.648 | 3.28x10^-3^ |
| HCC | 7.571 | 1.90x10^-5^ |
| HCC | 7.661 | 2.41x10^-5^ |
| HCC | 7.943 | 3.09x10^-3^ |
| CRC | 8.226 | 1.96x10^-6^ |
| CRC | 8.408 | 1.32x10^-2^ |
| HCC | 9.163 | 1.27x10^-4^ |
| HCC | 9.613 | 2.14x10^-6^ |
| HCC | 9.974 | 7.08x10^-5^ |
| CRC | **10.182** | 2.62x10^-7^ |
| CRC | 10.359 | 5.54x10^-7^ |
| CRC | 10.541 | 3.71x10^-2^ |
| HCC | 11.315 | 2.15x10^-2^ |
| CRC | **11.997** | 5.54x10^-7^ |
| HCC | 13.546 | 4.36x10^-3^ |
| HCC | 13.787 | 4.36x10^-3^ |
| HCC | 14.018 | 8.81x10^-3^ |
| HCC | 14.975 | 2.16x10^-7^ |
| HCC | 15.138 | 1.26x10^-5^ |
| HCC | 15.351 | 2.98x10^-4^ |
| HCC | 15.882 | 1.47x10^-4^ |
| HCC | 19.951 | 4.10x10^-8^ |
| HCC | 20.845 | 1.13x10^-5^ |
| HCC | 21.285 | 3.30x10^5^- |
| HCC | 22.276 | 3.88x10^-4^ |
| CRC | 23.166 | 2.54x10^-3^ |
| CRC | 23.801 | 8.83x10^-6^ |
| CRC | 24.807 | 9.09x10^-5^ |
| HCC | 28.003 | 4.85x10^-3^ |
| CRC | 32.045 | 4.45x10^-4^ |
| HCC | 41.781 | 4.05x10^-6^ |
| HCC | 51.559 | 2.37x10^-4^ |
| HCC | 54.396 | 1.13x10^-5^ |
| HCC | 55.435 | 1.98x10^-6^ |
| CRC | 68.188 | 1.16x10^-2^ |
| HCC | 82.182 | 3.30x10^-5^ |
| CRC | 118.336 | 6.08x10^-3^ |
| CRC | 172.524 | 5.85x10^-4^ |
