# Supplementary material for: Colon-Derived Liver Metastasis, Colorectal Carcinoma, and Hepatocellular Carcinoma Can Be Discriminated by the Ca2+-Binding Proteins S100A6 and S100A11
Source: PLoS One. 2008 Dec 2;3(12):e3767. doi: 10.1371/journal.pone.0003767 (PMC2585013; doi:10.1371/journal.pone.0003767)
Supplement: Table S4 — (0.04 MB DOC) [file pone.0003767.s005.doc]

Table S4: Significantly different signals which separate tissues from liver metastases derived from colorectal carcinoma (MTS) and colorectal carcinoma (CRC) detected on Q10 arrays.

| **Signal in** | **MW (kD)** | **P-value** |
| --- | --- | --- |
| CRC | 4.966 | 3.76x10^-2^ |
| MTS | 5.655 | 1.26x10^-2^ |
| CRC | 8.208 | 4.10x10^-4^ |
| CRC | 10.356 | 2.40x10^-3^ |
| MTS | 10.845 | 1.82x10^-3^ |
| MTS | 11.308 | 3.39x10^-2^ |
| CRC | 11.681 | 2.75x10^-2^ |
| CRC | 11.835 | 4.60x10^-2^ |
| MTS | 14.024 | 4.16x10^-2^ |
| CRC | 23.162 | 6.94x10^-3^ |
| MTS | 47.952 | 2.51x10^-2^ |
| MTS | 51.431 | 3.62x10^-3^ |
| MTS | 53.641 | 6.11x10^-3^ |
| MTS | 94.521 | 8.90x10^-3^ |
